# Properties of Allicin–Zein Composite Nanoparticle Gelatin Film and Their Effects on the Quality of Cold, Fresh Beef during Storage

**DOI:** 10.3390/foods12193713

**Published:** 2023-10-09

**Authors:** Ling Hu, Pengcheng Zhao, Yabo Wei, Xin Guo, Xiaorong Deng, Jian Zhang

**Affiliations:** 1 School of Food Science and Technology, Shihezi University, Shihezi 832003, China; huling0115@163.com (L.H.); zpc3049577959@163.com (P.Z.); 18935813163@163.com (Y.W.); guoxin24yjs@163.com (X.G.); dxr20099@163.com (X.D.); 2 Key Laboratory of Agricultural Product Processing and Quality Control of Specialty (Co-Construction by Ministry and Province), School of Food Science and Technology, Shihezi University, Shihezi 832003, China; 3 Key Laboratory for Food Nutrition and Safety Control of Xinjiang Production and Construction Corps, School of Food Science and Technology, Shihezi University, Shihezi 832003, China

**Keywords:** allicin, allicin–zein composite nanoparticles, gelatin film, fresh-keeping

## Abstract

Allicin is a kind of natural antimicrobial active substance, but its water solubility is poor, and it is easy to degrade. In order to improve the stability and bioavailability of allicin, allicin–zein composite nanoparticles (Al-Ze) were prepared by the combination method of antisolvent precipitation and electrostatic deposition, and their characteristic parameters, such as average particle size, polydispersity index (PDI), and ζ-potential, were analyzed. Then, Al-Ze was used as the delivery carrier for the active substance (allicin), and gelatin with good film-forming properties was selected as the film-forming matrix to prepare Al-Ze gelatin films. The optical properties, mechanical properties, and characterization parameters were used to analyze the prepared composite materials; the results confirmed that Al-Ze gelatin film has good mechanical properties and barrier properties. The prepared film was applied to the storage of cold, fresh beef, and the quality change of beef was monitored at 4 °C. The results showed that Al-Ze gelatin film could effectively delay the quality deterioration of beef. This paper provides a new idea and data support for the application of Al-Ze gelatin film in meat storage and fresh-keeping, and offers new insight for the promotion and application of allicin in the food industry.

## 1. Introduction

Allicin (diallylthiosulfinate) is a volatile organic sulfur compound extracted from allium plants in the allium family. Because allicin is such an effective thiol-trapping reagent, it has good broad-spectrum antimicrobial, anti-tumor, and antioxidant properties [1,2]. However, its properties are highly unstable, sensitive to temperature, oxygen, organic solvents, and pH, and prone to rapid decomposition, which limits its application and promotion. In recent years, nanoparticle technology based on embedding and delivery carriers has developed rapidly in the food industry. Based on this nano size, the Brownian motion of molecules within the system overcomes the influence of gravity and has dynamic stability [3], while these nanoparticles can optimize the inherent defects of bioactive substances [4,5]. Among them, zein, because it contains more than half of the hydrophobic amino acids, is insoluble in water, and is soluble in high concentration (55–90%) ethanol aqueous solution [6], can be formed into nanoparticles by antisolvent precipitation and self-assembly of bioactive substances to enhance the stability and availability of bioactive compounds. This makes it an ideal carrier for embedding and delivery of hydrophobic active compounds [7]. However, this system has high surface hydrophobicity, and exposed nanoparticles tend to aggregate in aqueous solutions; thus, one strategy to overcome its limitations is to add sodium caseinate to form a complex. Sodium caseinate contains a large amount of proline residues with a pyrrole ring structure, which are concentrated on the peptide chain, thus limiting the formation of ordered structures such as α-helix and S-lamella in the protein secondary structure. Due to the uneven distribution of residual water groups and residual groups and the aggregation of the initial 40–50 amino acids in the N-terminal, casein exhibits unique amphiphilic properties [8,9], which can modify the surface of zein nanoparticles and provide affinity through hydrogen bonding and hydrophobic interaction to improve the stability and encapsulation rate of the nanoparticles [10]. Among the various methods for preparing nanoparticles, the combination of antisolvent precipitation and electrostatic deposition has obvious advantages including a simple experimental process, low cost, and minimal equipment requirements [11], so it is often used to produce nanoparticles for various biopolymers [6]. The disadvantage is that the experiment requires the removal of alcohol, which increases the safety risk and the corresponding cost, and it cannot be used in non-alcoholic foods.

Gelatin is a polymer of peptide molecules. It is formed by multistage hydrolysis of collagen in animal skin, bone, muscle membrane, and other connective tissues and has good film-forming properties [12]. There are a large number of hydroxyl groups in the molecular structure of gelatin, in addition to many carboxyl groups and amino groups, which gives gelatin strong hydrophilicity. Gelatin is an important natural biomolecular material; due to its strong film-forming ability, good transparency and oxygen resistance, excellent renewable performance, good biodegradability, and low cost, it has a wide range of applications in food, pharmaceutical, chemical, and other fields. Studies have shown that natural antioxidant substances such as potassium sorbate and natural antimicrobial substances such as chitosan can be added to gelatin to prepare a gelatin film with antioxidant or antimicrobial effects; this can be wrapped on the surface of cold, fresh meat to reduce drip loss, improve color change, and extend shelf life [13,14]. Wu et al. [15] prepared a fish gelatin antibacterial composite film using cinnamaldehyde and its sulfobutyl ether-β-cyclodextrin complex, which effectively inhibited the growth of microorganisms in grass carp slices. At the end of storage, the total amount of total volatile base nitrogen (TVB-N) did not exceed 10 mg/100 g, indicating that the active coating could significantly extend the shelf life of fish. Yun et al. [16] extracted gelatin from salmon bone and used gallic acid/clove essential oil as the bioactive component to prepare a salmon bone gelatin–chitosan-based edible coating, and applied it to the refrigerated preservation of salmon fillets, extending the shelf life for at least 5 days. At the same time, from the perspective of sustainable development and environmental protection, gelatin is expected to replace traditional non-degradable packaging materials due to its natural source and biocompatibility, which has been favored by many researchers and consumers. On the other hand, the natural gelatin film is not very flexible, lacks elasticity and ductility, and has weak protection ability against microorganisms [17], so it is necessary to properly treat the gelatin film to better meet various preservation needs. This method of using gelatin as film-forming substrate, adding composite nanoparticles containing bioactive substances, and applying to the storage and preservation of cold meat can expand the application range of bioactive substances and provide a new idea for food packaging.

## 2. Materials and Methods

### 2.1. Materials

Allicin (analytically pure) and sodium caseinate (food grade) were purchased from Shanghai Maclin Biochemical Technology Co., Ltd., Shanghai, China. Zein (food grade) was purchased from Shanghai Ruixiang Biotechnology Co., Ltd., Shanghai, China. Gelatin (food grade) was purchased from Zhejiang Yinuo Biotechnology Co., Ltd., Lanxi, China. Fresh beef was purchased from the Jiuding Wholesale Market in Shihezi City. All reagents used in the experiment were analytically pure.

### 2.2. Preparation of Nanoparticles with Different Allicin Content

Al-Ze was prepared by the combination method of antisolvent precipitation and electrostatic deposition. We weighed and dissolved 0.4 g zein in 20 mL ethanol solution (*v*/*v* 80%), then added different concentrations of allicin (W_zein_/W_allicin_ 20:1, 10:1, 8:1, 5:1, 4:1), stirred with magnetic force for two hours (600 r/min), then added 80 mL of sodium caseinate solution (0.25%) and continued stirring evenly (600 r/min, 3 min). After rotating evaporation (40 °C, −0.1 MPa, 600 r/min, 15 min), the solution was centrifuged for 15 min (5000 r/min) to prepare composite nanoparticles loaded with different concentrations of allicin; they were labeled Al-Ze _(20:1)_, Al-Ze _(10:1)_, Al-Ze _(8:1)_, Al-Ze _(5:1)_, and Al-Ze _(4:1)_, and an Al-Ze _(20:0)_ complex without allicin was used as a blank control. The average particle size, PDI, and ζ-potential of the composite nanoparticles were determined by a laser particle size analyzer (Nano-ZS, Malvern Panalytical Instruments, Malvern, UK).

### 2.3. Preparation of Al-Ze Gelatin Films with Different Allicin Contents

We weighed 4.0 g gelatin and dissolved it in 100 mL pure water; it was then stirred (600 r/min) and dissolved with magnetic force at 60 °C for 30 min. We added 1.2 g of glycerol as a plasticizer (to weaken the hydrogen bonding force between molecules in the polymer), stirred with magnetic force to mix it evenly (600 r/min, 30 min), and added the nanoparticle dispersion liquid loaded with different concentrations of allicin prepared in Section 2.2, denoted as Al-Ze _(20:1)_ gelatin film, Al-Ze _(10:1)_ gelatin film, Al-Ze _(8:1)_ gelatin film, Al-Ze _(5:1)_ gelatin film, Al-Ze _(4:1)_ gelatin film, and blank control Al-Ze _(20:0)_. The solution was stirred with magnetic force (600 r/min) at 25 °C for 2 h, then degassed by ultrasonication for 30 min. A total of 25 ± 0.02 g of film forming liquid was taken into the mold for film casting, left for 5 h at 25 °C, and dried in a 50 °C constant temperature in a blast-drying oven for 10 h. Then, the film was peeled from the surface of the mold with tweezers and transferred to a constant temperature and humidity in a drying oven (25 °C ± 0.5 °C, RH: 54%) for 48 h for the determination and characterization of subsequent film properties.

### 2.4. Determination of Properties of Al-Ze Gelatin Films with Different Allicin Content

#### 2.4.1. Measurement of Film Thickness and Water Vapor Permeability Coefficient (WVP)

Five points were randomly selected on the composite nanofilm, and the thickness was measured with a digital micrometer (measuring range 0–25 mm, Wenzhou Weidu Electronics Co., Ltd., Wenzhou, China, accurate to 0.001 mm); the average value was taken as the final result.

A total of 10 mL of pure water was placed it in a clean weighing dish. The film was cut into appropriate sizes and used it to seal the weighing-dish mouth. Then the weighing dish was placed in the dryer and weighed once every 2 h, for a total of 6 times. The WVP of the film is calculated as Formula (1):

(1) $$\mathrm{WVP}=\frac{\Delta m\times d}{A\times t\times \Delta p}$$

where A is the area of the film, m^2^; t is the interval between two mass increments, h; Δm is the increased mass within time t, g; d is the thickness of the sample, mm; Δp is the vapor pressure difference between the two sides of the sample, 23.76 mmHg.

#### 2.4.2. Determination of Mechanical Properties of Films

With reference to the test method of Iwata et al, with slight modifications [18], tensile stress (TS) and elongation at break (EAB) of the film were measured by a texture analyzer (TA-TX plus, Stable Micro Systems, Godalming, UK). The calculation methods are shown in Formulas (2) and (3), respectively.

(2) $$\mathrm{TS}=\frac{F}{A}$$

where TS is the tensile strength, MPa; F is the maximum load, N; A is the cross-sectional area (width × thickness) of the film before the test, mm^2^.

(3) $$\mathrm{EAB}(\%)=\frac{L-L_{0}}{L_{0}}\times 100$$

where L_0_ is the original distance between the film samples, mm; L is the distance between the marks when the film sample breaks, mm.

#### 2.4.3. Determination of Moisture Content (MC), Swelling Property (SD), and Water Solubility (WS) of Films

We weighed the film sample (H_1_), dried it in an oven at 105 °C for 24 h, and weighed it again (H_2_). It was then transferred to a beaker containing 50 mL of distilled water, sealed and placed at 25 °C for 24 h, and the weight was taken again (H_3_) after drying. It was then re-placed in the oven at 105 °C and dried for 24 h and then weighed (H_4_). The MC, SD, and WS of the composite film are calculated using Formulas (4)–(6), respectively:

(4) $$\mathrm{MC}(\%)=\frac{H_{1}-H_{2}}{H_{1}}\times 100$$

(5) $$SD(\%)=\frac{H_{3}-H_{2}}{H_{2}}\times 100$$

(6) $$\mathrm{WS}(\%)=\frac{H_{2}-H_{4}}{H_{2}}\times 100$$

#### 2.4.4. Determination of Optical Properties of Films

On the films with different allicin content, five points were randomly selected by a handheld colorimeter (SC-10, Shenzhen Threenh Technology Co., Ltd., Shenzhen, China) to measure the chromatic aberration of the films, and the average of the results were taken. The opacity was determined according to the method of Jongjareonrak et al. [19]. The prepared film was cut into a rectangular strip of 10 mm × 40 mm and pasted on the smooth side of the inner side of the cuvette. The optical properties of gelatin films were determined by UV and visible wavelength scanning at 200–400 nm and 400–800 nm, respectively, with a blank test tube as a control.

### 2.5. Characterization of Al-Ze Gelatin Films with Different Allicin Content

#### 2.5.1. Fourier Transform Infrared Spectroscopy (FTIR)

FTIR (Bruker Vertex 70v Prior, Karlsruhe, Germany) was used to test the infrared spectrum of the film in the range of 4000 cm^−1^ to 400 cm^−1^ in the attenuated total reflection mode, with a resolution of 4 cm^−1^ and totaling 16 scans. The chemical structures of films with different allicin content were characterized.

#### 2.5.2. Microstructure Observation

The smooth and evenly distributed parts of the film were selected (2 mm × 2 mm) and fixed on the sample stage with conductive adhesive; then, we observed the surface and cross-section microstructure of the film samples after vacuum using an SEM (SU8010, Hitachi Scientific Instruments Co., Ltd., Beijing, China).

#### 2.5.3. Thermogravimetric Analysis (TG)

The 5 mg film sample was weighed and placed in a thermogravimetric analyzer (STA 449F5, NETZSCH Instruments, Selb, Germany) and measured at 30–600 °C at a heating rate of 20 °C/min; the thermal properties were analyzed.

### 2.6. Effect of Al-Ze Gelatin Film on the Quality of Cold, Fresh Beef during Storage

#### 2.6.1. Sample Processing

After removing excess fat and fascia, fresh beef was divided into 60.0 g/part, and Al-Ze gelatin films with an allicin content of 0.04 g and 0.08 g were prepared according to Section 2.3, labeled as Al-Ze _(10:1)_ gelatin film and Al-Ze _(5:1)_ gelatin film. Pure gelatin films were prepared, labeled as Al-Ze _(0:0)_ gelatin film, and the beef was completely wrapped in film and then put into self-sealing bags for sealing. Beef samples without any treatment were used as the control group (CK), and all of the above were refrigerated at 4 ± 1 °C. The samples were taken out every 2 days over 12 days to determine pH value, color, mass loss rate, TVB-N, thiobarbituric acid (TBA) value, and aerobic plate count (APC) and for sensory evaluation.

#### 2.6.2. Analysis of Color during Storage

Three points were randomly selected on each group of beef samples. Their color during storage was measured by a colorimeter, and the average value was taken as the final result. L* represented brightness, a* represented redness and greenness, and b* represented a yellow-blue color.

#### 2.6.3. Analysis of pH Value during Storage

This experiment was conducted in accordance with GB 5009.237-2016 [20]. Due to the change in the concentration of hydrogen ions in the sample, the potential difference between the indicating electrode and the reference electrode in the pH meter was generated, and the signal was amplified and converted to achieve the purpose of pH value measurement.

#### 2.6.4. Analysis of Mass Loss Rate during Storage

The initial weight of each group of beef samples was accurately weighed and recorded as M_1_. They were then sealed in a self-sealing bag after treatment and stored at 4 ± 1 °C. The samples were weighed every 2 days, recorded as M_2_; before weighing, filter paper was used to absorb the juice on the surface of the sample. The mass loss rate can be calculated using following Formula (7):

(7) $$\mathrm{Mass}\;\mathrm{loss}\;\mathrm{rate}(\%)=\frac{M_{1}-M_{2}}{M_{1}}\times 100$$

#### 2.6.5. Analysis of Total Volatile Base Nitrogen (TVB-N) during Storage

For the determination of TVB-N, refer to GB 5009.228-2016 [21]. Based on the principle of semi-trace nitrogen determination, the content of TVB-N was determined using an automatic Kjeldahl nitrogen analyzer (Kjeltec 8400, ROSS Sweden).

#### 2.6.6. Analysis of Thiobarbituric Acid (TBA) Value during Storage

The analysis was based on the method of Wei [22], with slight modifications. We measured the absorbance value of the sample at a wavelength of 532 nm using an ultraviolet-visible spectrophotometer (T3200, Shanghai Youke Instrument Co., Ltd., Shanghai, China). We calculated the TBA (mg MDA/kg) value, expressed as the equivalent of the malondialdehyde content, as shown in Formula (8):

(8) $$\mathrm{TBA}=\frac{A_{532\mathrm{nm}}}{m}\times 9.48$$

where A_532nm_ is the absorbance of the solution; m is the mass of the beef sample, g; 9.48 is a constant.

#### 2.6.7. Analysis of Aerobic Plate Count (APC) during Storage

According to the method of GB4789.2-2022 [23], the total number of microbial colonies formed in each g of the tested sample was obtained by treating the sample and cultivating it under certain conditions (36 ± 1 °C).

#### 2.6.8. Sensory Evaluation during Storage

The sensory analysis was conducted in accordance with the national standard, with slight modifications; 10 professionally trained personnel were selected for sensory evaluation. The sensory evaluation standards are shown in Table 1.

### 2.7. Statistical Analysis

All tests were carried in triplicate. SPSS 25.0 statistical software was used to analyze the experimental data, and the Duncan method was used to perform multiple comparison tests (*p* < 0.05). Data were presented as mean ± standard deviation.

## 3. Results and Discussion

### 3.1. Average Particle Size, PDI, and ζ-Potential Analysis of Al-Ze Nanoparticles

**Figure 1:**
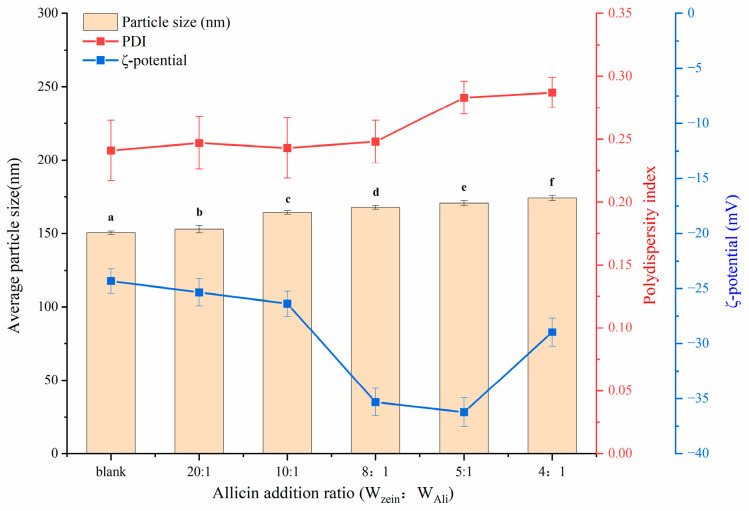
Changes of particle size, PDI, and ζ-potential of nanoparticles with different allicin content. (a–f): different lowercase letters denote significant differences (*p* < 0.05).

From Figure 1, it can be seen that with the increase of allicin content, the average particle size of the nanoparticles gradually increases, but the PDI has no significant change and is below 0.3, indicating that despite the gradual increase of allicin content, the nanoparticle dispersion is still relatively uniform, showing good stability. Davidov Pardo et al. encapsulated resveratrol in zein and also obtained a nanoparticle system with good stability [24]. *ζ*-potential is one of the important indexes used to measure the stability of colloidal dispersion systems. The higher the *ζ*-potential, the stronger the electrostatic repulsion between particles, which can effectively prevent the precipitation of nanoparticles in colloidal solution. Previous studies have claimed that the absolute value of *ζ*- potential greater than 30 mV can make the system more stable [25]. When the ratio of zein to allicin was 8:1 and 5:1, the corresponding *ζ*-potential was −35.32 mV and −36.23 mV, respectively, with higher absolute potentials, indicating that the colloidal dispersion solution formed under the corresponding conditions was more stable and had a lower possibility of aggregation.

### 3.2. Analysis of Properties of Nanoparticle Films with Different Allicin Content

#### 3.2.1. Analysis of Thickness, WVP, and Mechanical Properties

The thickness of the films in each group is shown in Table 2; the thickness of the Al-Ze _(20:0)_ gelatin film is 0.125 nm. With the increase of allicin content, the interaction force between molecules increases, and the thickness of the films increases, but the increase is small. This may be due to the increase in components within the system, which disrupts the ordered structure of the film due to interactions, leading to changes in the solid content of the film matrix during the film formation process, thereby changing the thickness of the film [26]. In Khedri et al.’s study of casein phosphopeptide gelatin films, a similar pattern was observed, where the film thickness increased with the increase of active substance concentration [27].

Generally, gelatin films have high water vapor transmission rates. Studies have shown that the WVP of gelatin film is 5.68 × 10^−11^ g·cm/cm^2^·s·Pa, while the WVP of Al-Ze _(20:0)_ gelatin film is 5.37 × 10^−11^ g·cm/cm^2^·s·Pa. This may be because the hydrophobic zein restricts the diffusion of water on the surface of the film, thereby increasing the intermolecular force of the film polymer chain, reducing the chain fluidity, reducing the free volume, and ultimately increasing the hydrophobicity of the film. Taghizadeh et al. observed the same result when using photosensitizer-induced cross-linking to improve the physicochemical properties of gelatin films [28]. The interaction between substances could reduce the hydrophilic functional groups in the membrane matrix and increase the hydrophobic amino acids on the membrane surface, resulting in changes in the WVP of the membrane. At the same time, nanoparticles can occupy the pores of the film matrix and form a denser network structure, which creates a tortuous path for water molecules, thus hindering the diffusion of water vapor and improving the barrier performance of the film [9]. With the increase of allicin concentration, the WVP value of the Al-Ze _(4:1)_ gelatin film decreases to 4.52 × 10^−11^ g·cm/cm^2^·s·Pa, the hydrophobicity is further enhanced, and the water resistance of the film is improved, which all play an important role in the modification of gelatin film.

Mechanical strength is the key parameter when evaluating the properties of films. Generally, gelatin films do not have excellent mechanical strength; good mechanical strength is the basis for the promotion and application of edible films, which limits the scope of application of gelatin films. From Table 2, with the increase of allicin content, the TS and EAB of the film both showed an increasing trend, increasing from 19.45 MPa to 25.76 MPa and 31.97% to 71.22%, respectively. The mechanical strength improved significantly. This may be due to the hydrophobic properties of allicin nanoparticles, which improve the flexibility of gelatin films. Hou et al. drew similar conclusions in the study of the properties of chitosan-SiO_2_ nanoparticle starch film. Due to the small size and large specific surface area of nanoparticles, which can be associated with biopolymers through hydrogen bonds and interface interactions, they increase the strength of the film [29]. With the increase of allicin content, the nanoparticles and gelatin formed a suitable ratio, and the interaction between the two formed a uniform three-dimensional network structure, which enhanced the interface adhesion [30,31], thus improving mechanical properties.

#### 3.2.2. MC, SD, and WS Analysis

Pure gelatin film has poor water resistance and high moisture content [12,32]. As shown in Table 3, with the increase of the concentration of allicin, the MC of the film showed a decreasing trend. For edible films, having a certain water resistance can block the migration of water in the packaged food, thus extending the shelf life of the food. There is an important relationship between the MC and SD of the films. The data show that the SD of the films decreases significantly with the increase of allicin concentration. This may be because the addition of nanoparticles enhanced the hydrophobicity of the film, reduced the number of hydrophilic groups in the film, enhanced the interaction between molecules, formed a dense structure, and reduced the SD of the film. At the same time, its WS decreases further, which has the same change trend as the MC and SD. Jiang et al. drew similar conclusions in the development of zein edible films containing different catechin/cyclodextrin metal-organic frameworks. The addition of fillers reduced the MC, SD, and WS of the films to varying degrees [33].

#### 3.2.3. Optical Property Analysis

**Figure 2:**
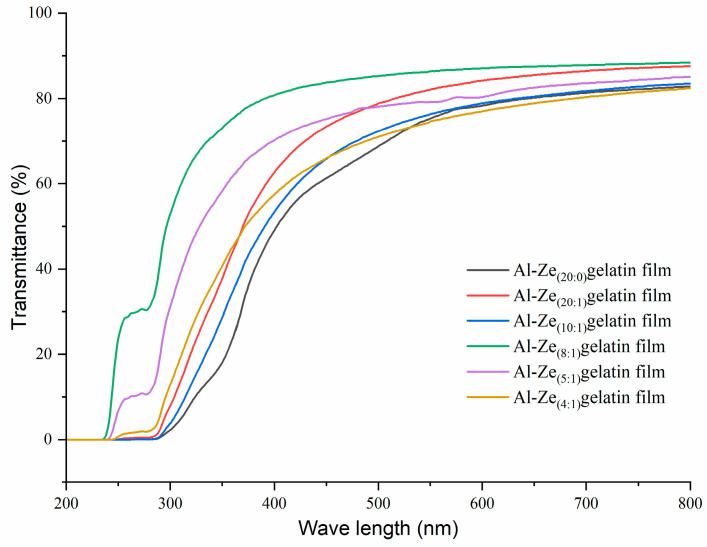
Light transmittance of nanoparticle gelatin films with different allicin content.

Transparency is of great significance to edible films and directly affects the color of the films [19]. As can be seen from Figure 2, with the increase of allicin concentration, the transmittance of the film in the ultraviolet and visible regions gradually increased, among which the transmittance of the Al-Ze _(8:1)_ gelatin film was the largest, due to the high content of hydrophobic amino acids in zein being conducive to the compatibility and dispersion of allicin in the solid polymer matrix, thus increasing the transparency of the film [34]. However, with the further increase of allicin concentration, its transmittance gradually decreased, which was related to the increase of the b*, possibly because allicin itself is light-yellow, which had a certain impact on the transmittance [35]. Sara et al. found in the development of gelatin films incorporated with casein phosphopeptides that the light transmittivity of the film decreased with the increase of the concentration of bioactive casein phosphopeptides [27].

Table 4 shows the chromatism of the film, expressed in L*, a*, and b* values. L* is the brightness, a* is red, ranging from green (−) to red (+), and b* is yellow, ranging from blue (−) to yellow (+). With the increase of allicin concentration, the L* of the film gradually decreased, while the a* and b* showed a gradual upward trend, with a* changing from negative to positive and b* rising from 6.02 to 8.74. Since allicin is light-yellow, it may have played a certain role in the color of the film, resulting in a gradual increase in the yellowness value of the film.

#### 3.2.4. Analysis of Structure

(1) FTIR

**Figure 3:**
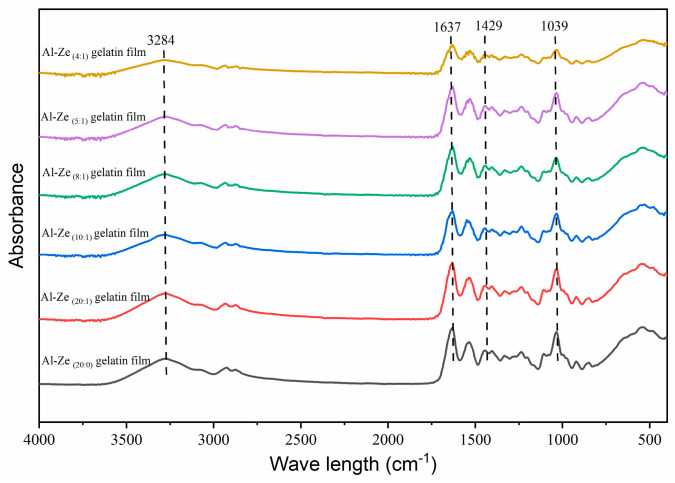
ATR-FTIR images of nanoparticle gelatin films with different allicin contents.

FTIR was used to analyze the molecular interactions among various components in Al-Ze gelatin films. As shown in Figure 3, the Al-Ze gelatin films of each group have similar ATR-FTIR spectra, indicating that they possess extremely similar functional groups. The peak located near the wavelength of 3284 cm^−1^ indicates the stretching vibration of N-H and O-H groups [36], where the intensity of the peak increases gradually with the increase of allicin content in the nanoparticles, which may be due to the intermolecular hydrogen bond between the hydroxyl group in Al-Ze and the phenolic hydroxyl group in the gelatin molecule. Ji et al. and Davidov-Pardo et al. also reached a similar conclusion, that hydrogen bonds are involved in complex formation and may be one of the main binding forces between substances [24,37]. In addition, the amide I region formed by the film at 1637 cm^−1^ is mainly coupled with COO by the C=O tensile vibration and hydrogen bond; the absorption band located near 1429 cm^−1^ can be attributed to O-H bending [38]. The spike observed at 1039 cm^−1^ represents the interaction between O-H groups in glycerol and gelatin molecules, indicating that Al-Ze successfully mixed with the film, forming a matrix of gelatin and glycerol, which is similar to the results of Jiang et al. [33].

(2) Microstructure

**Figure 4:**
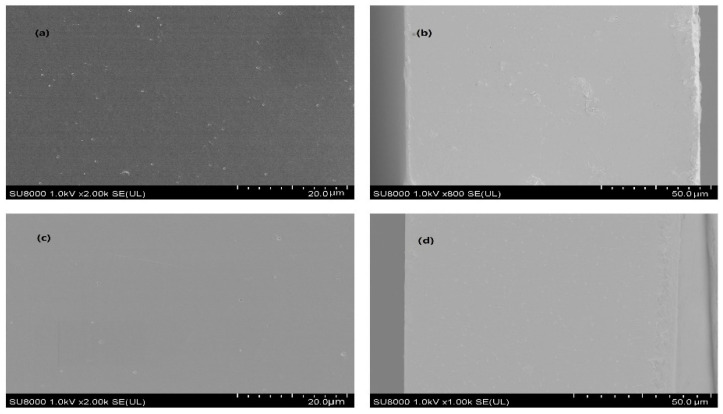
SEM images of nanoparticle gelatin films with different allicin contents. (**a**,**c**) are the surface images of the Al-Ze _(20:0)_ gelatin film and Al-Ze _(10:1)_ gelatin film respectively; (**b**,**d**) are cross-sectional images of the Al-Ze _(20:0)_ gelatin film and Al-Ze _(10:1)_ gelatin film respectively.

Figure 4 shows the micromorphologies of nanoparticle gelatin films with different allicin content observed by SEM under an accelerating voltage of 1 kV, where (a) and (c) are the surface images of the Al-Ze _(20:0)_ gelatin film and Al-Ze _(10:1)_ gelatin film when magnified 2000 times, respectively; (b) and (d) are cross-sectional images of the Al-Ze _(20:0)_ gelatin film and Al-Ze _(10:1)_ gelatin film at 800× and 1000× magnification, respectively. It can be seen from (a) that there are many white particles on the surface of the Al-Ze _(20:0)_ gelatin film, which may be sodium caseinate with an agglomeration effect, indicating that slight phase separation may have occurred inside the Al-Ze _(20:0)_ gelatin film; in (b), the formed film has an uneven texture distribution and irregular grooves in the cross-sectional structure, which may be caused by the irregular accumulation of gelatin molecules. In (c) and (d), it can be seen that after adding allicin, the white particles and grooves of the composite film disappeared, and the surface and cross-sectional structure of the film became regular and dense, indicating that the gelatin has good compatibility with allicin. The formation of these structures is attributed to the hydrogen bond and interpenetrating network structure between allicin and gelatin, and the results of SEM further explain the improved mechanical properties of Al-Ze gelatin films. Similarly, Kchaou et al. studied the microstructure of the film composite of cuttlefish protein hydrolysate and gelatin, and the microphotographs showed that it had a good interaction and uniform structure [39].

(3) TG analysis

**Figure 5:**
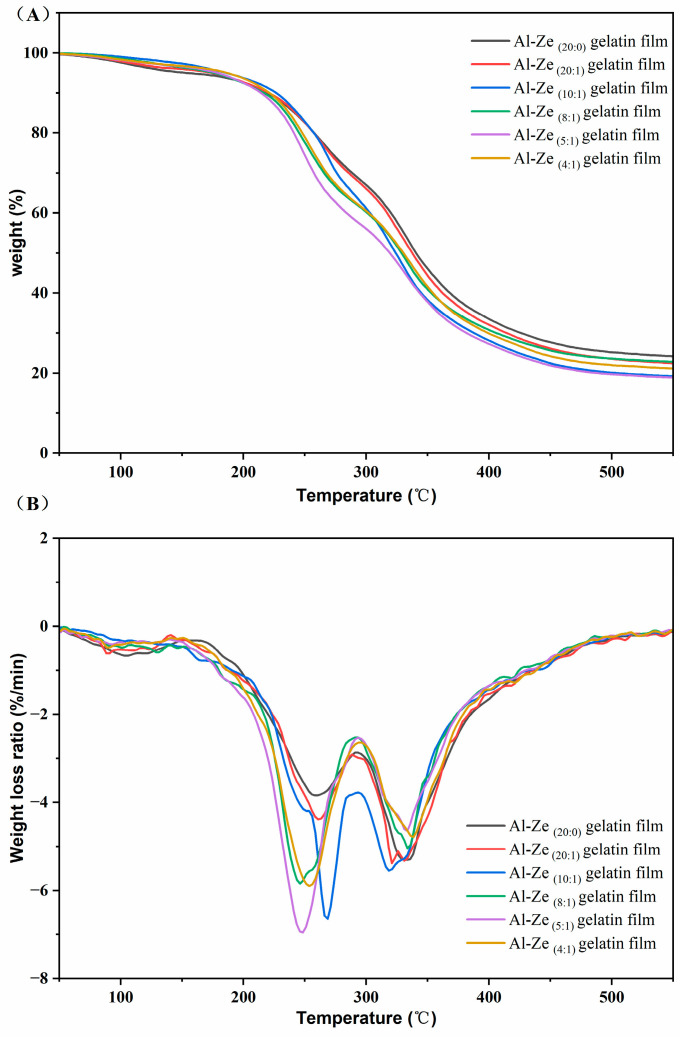
TG and DTG of nanoparticle gelatin films with different allicin contents. Where (**A**) is the TG curve of nanoparticle gelatin films with different allicin content, (**B**) is the differential thermogravimetric (DTG) curve of nanoparticle gelatin films with different allicin contents.

As shown in Figure 5A is the TG curve of nanoparticle gelatin films with different allicin content, indicating the relationship between the weight loss and temperature of the gelatin film. With the increase of temperature, the mass loss of the films in different thermal degradation stages showed a similar trend; this shows that the content of allicin has no significant effect on the TG of the composite films. Figure 5B shows the differential thermogravimetric (DTG) curve of nanoparticle films with different allicin contents, indicating the relationship between the change rate of weight loss of gelatin films and temperature. The thermal degradation of the film can be divided into three stages: the first stage (50–150 °C), which may be due to the evaporation of water in the gelatin film and the volatilization of some low-molecular-weight compounds led to the mass loss at this stage. In the second stage (150–400 °C), the film has a relatively obvious degradation, and the weight change is most obvious. This stage is the process of nanoparticle decomposition. Compared with the Al-Ze _(20:0)_ gelatin film, the thermal degradation temperature of the film containing allicin is lower, which may be due to the interaction between Al-Ze and the groups of gelatin molecules [40]. In the range of 150–300 °C, the weight loss of films decreased most obviously, and the change of weight loss of films with different allicin content was not the same, which may be because the different concentration of allicin led to the difference in the force between nanoparticles and gelatin. When the temperature is 300–400 °C, the mass loss rate of the films changes in the same trend, where the mass loss rate of the Al-Ze _(5:1)_ gelatin film decreases the fastest and changes the most significantly. The third stage occurs at 400–500 °C, which is the thermal decomposition process of glycerol.

### 3.3. Effects of Al-Ze Gelatin Film on the Quality of Chilled Beef during Storage

#### 3.3.1. Change in Color

**Figure 6:**
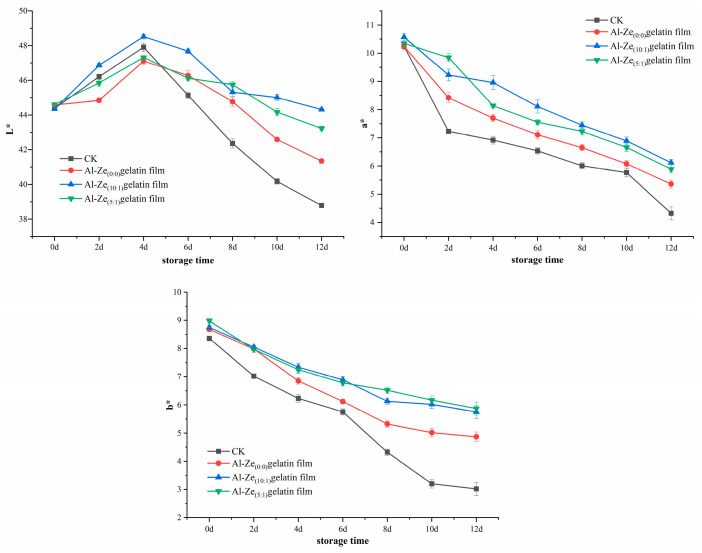
Changes in color during storage.

In daily production and life, color is an important standard for consumers to judge whether meat products are fresh and can be purchased [41]. L* reflects the brightness of the beef, a* reflects the red-green value, b* reflects the yellow-blue value, in which the content of myoglobin in the beef determines the size of a*. Myoglobin in the meat is oxidized by the air to produce oxygenated myoglobin; if it continues to be exposed to the air, it will be further oxidized to high ferrimyoglobin, thus making the beef dark brown. As can be seen from Figure 6, the L* of fresh beef is 44.36, a* is 10.35, and b* is 8.68. During the preservation of beef, the L* values of each group of samples showed a trend of temporary increase and then continuous decrease; with the extension of storage time, the surface water of beef is evaporated and the brightness is reduced [42]. Compared with the control group, allicin can reduce the hydrolysis of protein in meat, reduce the exposure of hydrophobic groups and water loss, change the refractive index of the surface of beef, and effectively prevent the beef from turning white. In addition, a* and b* showed a continuous downward trend. Among them, the decline rate of the CK group is the fastest. On the 12th day of beef storage, the L* of the CK group decreased to below 40, a* to 4.32, and b* to 3.02. For the Al-Ze _(0:0)_ gelatin film, the L* is 41.35, a* is 5.36, and b* is 4.87. While the L* of the Al-Ze _(10:1)_ gelatin film and Al-Ze _(5:1)_ gelatin film remained around 44, the a* decreased but remained around 6.11, and b* was near 5.75; the color was obviously better than that the Al-Ze _(0:0)_ gelatin film and CK groups, indicating that adding allicin could effectively delay the decline of a* and b* in the beef samples during storage, maintain the brightness and redness of beef samples, and improve the color stability. Similarly, Giselle et al. applied chitosan-gelatin film to the retail preservation of steak and found that the stability of steak color was improved [43].

#### 3.3.2. Changes in pH Value

**Figure 7:**
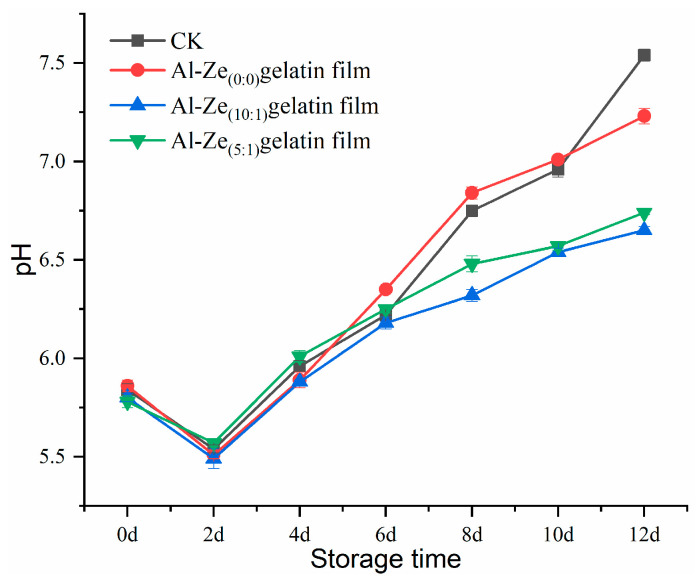
Changes in pH during storage.

pH value can affect the color appearance, water retention capacity, and bacterial growth and reproduction of beef during storage [44]. Figure 7 shows the changes in pH value of beef in each group during storage at 4 °C. It can be seen from the figure that the pH value of samples in all groups dropped to about 5.5 within two days, which may be due to the glycolysis of muscle cells of beef after slaughter, and the production of acidic substances such as phosphoric acid and lactic acid by glycogen and muscle-phosphate, which caused the pH value of beef to decline and fall into the acidic range [45]. Within 2 to 12 days, with the extension of storage time, the pH value of beef in all groups showed an obvious rising trend. Ding et al. reached a similar conclusion in their study on the quality of pork during storage. This is mainly because spoilage microorganisms and endogenous enzymes break down the proteins in beef, producing ammonia, bioamines, and other alkaline substances [46]. Both the CK group and Al-Ze _(0:0)_ gelatin film maintained first-order freshness within 2 to 6 days, with the pH value greater than 6.7 on the eighth day, indicating that the shelf life of cold, fresh beef is less than 8 days. However, the Al-Ze _(10:1)_ gelatin film and Al-Ze _(5:1)_ gelatin film had second level freshness on the eighth day and still did not reach the spoilage threshold when the storage time was extended to 12 days, which fully indicates that the Al-Ze gelatin film had a good preservation effect.

#### 3.3.3. Change in the Rate of Mass Loss

**Figure 8:**
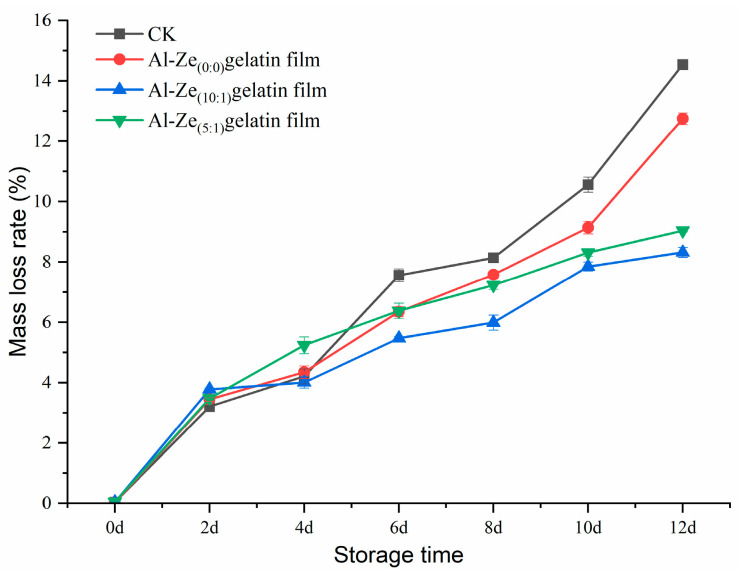
Changes in rate of mass loss during storage.

The mass loss of meat is mainly due to the juice loss and the destruction of endogenous enzymes in muscle tissue, resulting in the loss of nutrients and affecting the final quality of the product [47]. As can be seen from Figure 8, the mass loss rate of beef samples in each group showed a significant upward trend during storage at 4 °C, among which the mass loss rate of the CK group decreased most significantly, reaching 14.54% on the 12th day. The mass loss rate of the Al-Ze _(10:1)_ gelatin film and Al-Ze _(5:1)_ gelatin film was relatively low, between 8.32 and 9.04%, which may be because the film coating treatment leads to a certain degree of moisture retention, delays the drip loss, and reduces the quality loss rate of beef. Meanwhile, due to the antibacterial and antioxidant effects of allicin, it can inhibit the growth and reproduction of microorganisms, prevent the degeneration of beef protein, and effectively reduce the loss of water. Giselle et al. applied chitosan-gelatin film to the retail preservation of steak, which significantly reduced the mass loss of steak and extended its shelf life [45].

#### 3.3.4. The Change of total Volatile Base Nitrogen (TVB-N)

**Figure 9:**
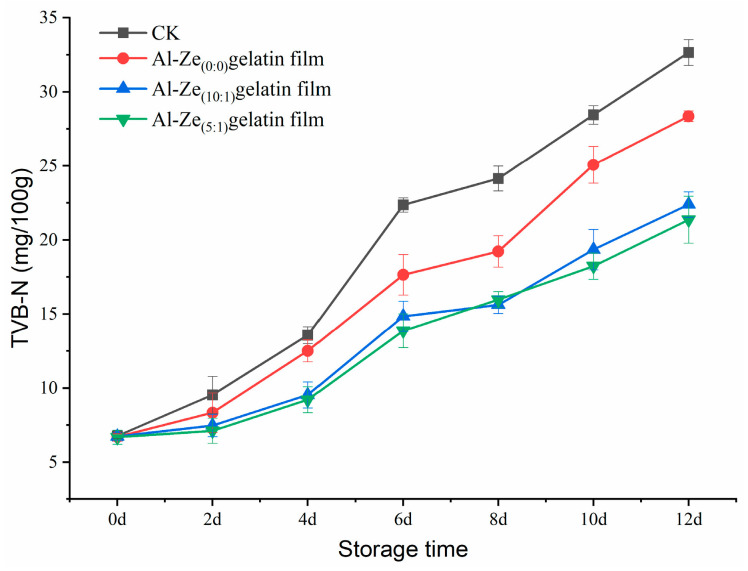
Changes in TVB-N during storage.

TVB-N is the product of decomposition of protein by bacteria and enzymes in the process of spoilage of animal food and can be used to judge the freshness of meat [48]. Figure 9 shows the changes of TVB-N in beef during storage at 4 °C. It can be seen from the figure that with the extension of storage time, the content of TVB-N in all groups showed an increasing trend, and the content of TVB-N in the CK group increased faster. When stored to the sixth day, the TVB-N content of the CK group was 22.36 mg/100 g, and the Al-Ze _(0:0)_ gelatin film content was 17.65 mg/100 g, which exceeded the acceptable threshold of TVB-N in fresh meat stipulated by the national standard of 15 mg/100 g [49]. At this time, the Al-Ze _(10:1)_ gelatin film and Al-Ze _(5:1)_ gelatin film were lower than 15 mg/100 g, and the beef had not yet deteriorated. This may be because the dense structure of the film itself has the effect of insulating oxygen and water to a certain extent; at the same time allicin can delay the lipid oxidation process and achieve the effect of extending the storage period of cold, fresh beef.

#### 3.3.5. The Change of Thiobarbituric Acid (TBA)

**Figure 10:**
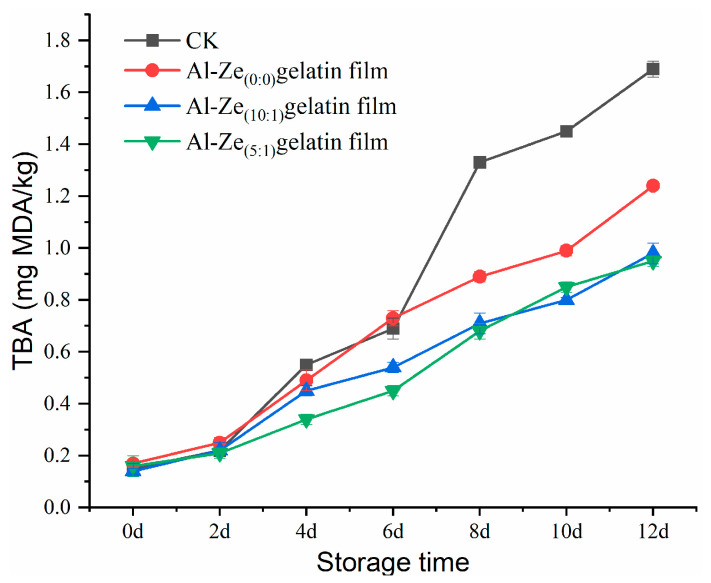
Changes in TBA during storage.

In existing studies, the TBA content is commonly selected to analyze the lipid oxidation degree of meat products, as an important indicator to evaluate the degree of rancidity in meat products. As shown in Figure 10, the TBA content of the CK group was 0.143 mg MDA/kg. With the extension of storage time, the TBA content in all groups showed an increasing trend, but with varying increases. At the sixth day of storage, the TBA content of the CK group was 0.694 mg MDA/kg, which exceeded the critical value of 0.664 mg MDA/kg for sub-fresh meat stipulated by the national standard. When stored to the eighth day, the TBA content of CK exceeded 1.000 mg MDA/kg, which belongs to the category of spoiled meat as stipulated by the national standard. After 10 days of storage, the TBA content of the CK group and Al-Ze _(0:0)_ gelatin film were both greater than 1.000 mg MDA/kg, indicating that the beef samples had completely deteriorated. Only on the 12th day of storage did the TBA content of the Al-Ze _(10:1)_ gelatin film and Al-Ze _(5:1)_ gelatin film approach the critical standard for spoiled meat. The results indicated that Al-Ze film coating could effectively slow down the increase of TBA content in beef samples and inhibit the lipid oxidation rate of chilled beef. Alizadeh-Sani et al. applied nano-structured active packaging with TiO_2_ and essential oils to the preservation of refrigerated meat. The results showed that active packaging significantly reduced lipid oxidation and lipolysis of mutton during storage, extending its shelf life by 9 days [50].

#### 3.3.6. Change in Aerobic Plate Count (APC)

**Figure 11:**
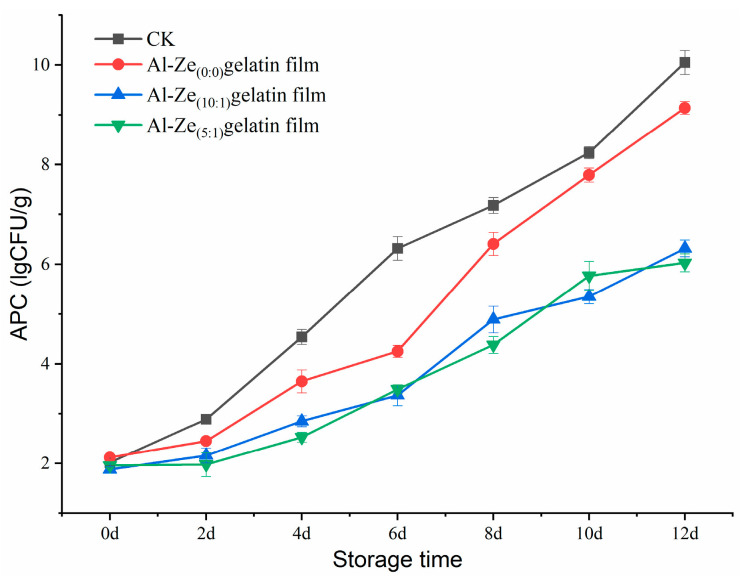
Changes in APC during storage.

APC is an important index to evaluate the freshness and quality of beef [51]. As shown in Figure 11, during storage at 4 °C, the APC in samples of all groups showed a significant increasing trend. On the eighth day of storage, the total number of CK and Al-Ze _(0:0)_ gelatin film colonies were greater than 6.0 lg (CFU/g), which exceeded the maximum acceptable range of the total number of colonies in fresh meat as stipulated by the national standard, indicating that the single gelatin film coating had no obvious effect on the preservation of cold, fresh beef. The total colonies of the Al-Ze _(10:1)_ gelatin film and Al-Ze _(5:1)_ gelatin film reached the spoilage threshold on the 12th day of storage, at 6.32 lg (CFU/g) and 6.02 lg (CFU/g), respectively. This proves that Al-Ze gelatin film coating can effectively inhibit the growth and reproduction of microorganisms in beef. Research has shown that allicin can react with cysteine in protein to produce S-allylmercapto-adducts, thus inhibiting the enzymes necessary for microbial growth and metabolism [52]. It can also destroy the tissue conformation of the protein, so that the protein loses its signal transduction function, thus playing an antimicrobial role. Similarly, Cui et al. applied tea tree essential oil liposome/chitosan nanofiber membranes to chicken samples, and the results showed good antibacterial activity, effectively maintaining the quality of chicken meat within 4 days of storage [53].

#### 3.3.7. Sensory Evaluation

**Figure 12:**
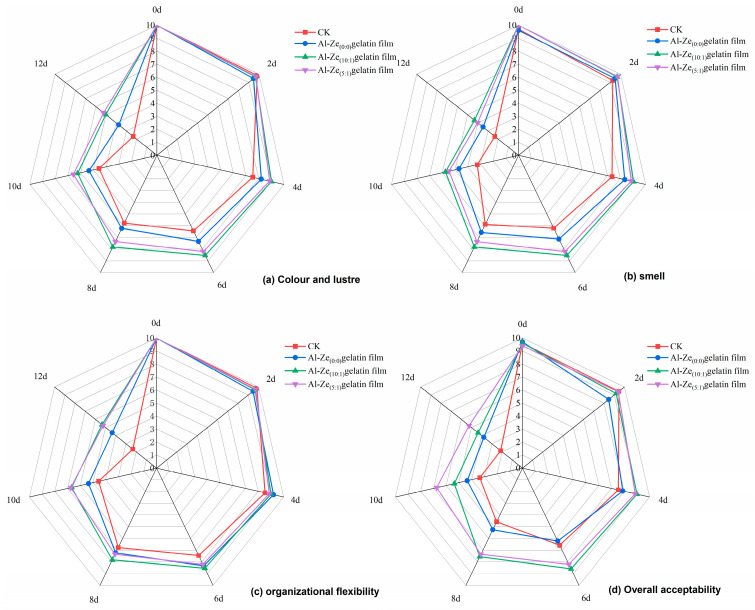
Sensory changes during storage.

The sensory score is one of the intuitive indicators to judge the overall quality of beef. As can be seen from Figure 12, for different storage periods of beef, the sensory scores of each group gradually decreased with the extension of storage time. When the storage time was from 0 to 6 days, the sensory score of the CK group decreased the most. From day 6 onwards, the sensory scores of the CK group and Al-Ze _(0:0)_ gelatin film declined rapidly, indicating that the degree of spoilage of beef samples increased significantly. On the eighth day of storage, the sensory score of the Al-Ze _(0:0)_ gelatin film dropped below 6, and the smell and color changed significantly; both were no longer acceptable to the evaluators. When the Al-Ze _(10:1)_ gelatin film and Al-Ze _(5:1)_ gelatin film were stored on the 10th day, the score dropped to 6 points, which may be due to the gradual deterioration of the beef; allicin has a certain antimicrobial effect, which can delay the spoilage of beef through the slow release of the film. In conclusion, Al-Ze gelatin film has a significant positive effect on the sensory quality of beef and can significantly improve the sensory scores of evaluators, further supporting the relevant physicochemical indexes of the above-mentioned experiment.

## 4. Conclusions

In the present study, Al-Ze was added to gelatin to prepare Al-Ze gelatin film. By measuring the characteristic parameters of the film, it was proven that its mechanical properties, water resistance, thermal stability, and uniformity were significantly improved. Furthermore, Al-Ze gelatin film with different allicin content was applied to analyze the refrigeration and fresh-keeping of beef. By measuring the quality change parameters of beef samples, it was found that Al-Ze gelatin film effectively delayed the quality deterioration of beef during storage, which provides a new method for meat preservation. This study also concluded that Al-Ze is an effective carrier for the delivery of the bioactive substance allicin, and the developed Al-Ze gelatin film has application prospects in food packaging.

## Figures and Tables

**Table 1:** Sensory evaluation criteria for chilled fresh beef.

	Color and Luster	Smell	Tissue Elasticity	Overall Acceptability
Very good (9–10 points)	Bright red, shiny	Fresh beef smell, no odor	Good elasticity, immediate recovery of dents after finger pressure	Acceptable
good (7–8 points)	Bright red, matte	No obvious odor	Good elasticity, the depression can be restored after finger pressure	Basically acceptable
not good (4–6 points)	Dark red in color, matte	The odor is strong and obvious	Poor elasticity, not easy to recover after finger pressure	Difficult to accept
very poor (0–3 points)	Dark brown, matte	Strong odor, unacceptable	Poor elasticity, unable to recover after finger pressure	unacceptable

**Table 2:** Thickness, WVP, TS, and EAB of nanoparticle gelatin films with different allicin content.

Film Sample	Thickness (mm)	WVP (10^−11^ g·cm/cm^2^·s·Pa)	TS (MPa)	EAB (%)
Al-Ze _(20:0)_ gelatin film	0.125 ± 0.003 ^a^	5.37 ± 0.14 ^d^	19.45 ± 0.26 ^a^	31.97 ± 2.03 ^a^
Al-Ze _(20:1)_ gelatin film	0.150 ± 0.006 ^bc^	5.24 ± 0.10 ^d^	23.93 ± 0.49 ^b^	39.92 ± 2.82 ^a^
Al-Ze _(10:1)_ gelatin film	0.141 ± 0.009 ^b^	5.21 ± 0.12 ^cd^	25.85 ± 0.35 ^b^	51.19 ± 2.86 ^b^
Al-Ze _(8:1)_ gelatin film	0.151 ± 0.007 ^c^	5.00 ± 0.04 ^c^	25.24 ± 0.64 ^c^	55.87 ± 6.35 ^b^
Al-Ze _(5:1)_ gelatin film	0.150 ± 0.003 ^bc^	4.74 ± 0.09 ^b^	25.28 ± 0.36 ^c^	58.97 ± 3.52 ^b^
Al-Ze _(4:1)_ gelatin film	0.156 ± 0.006 ^c^	4.52 ± 0.05 ^a^	25.76 ± 0.67 ^c^	71.22 ± 3.38 ^c^

Note: Water vapor permeability coefficient (WVP), tensile stress (TS), and elongation at break (EAB). ^a–d^: different lowercase letters denote significant differences (*p* < 0.05).

**Table 3:** MC, SD and WS of nanoparticle gelatin films with different allicin content.

Film Sample	MC (%)	SD (%)	WS (%)
Al-Ze _(20:0)_ gelatin film	30.62 ± 0.92 ^e^	31.75 ± 0.45 ^a^	30.01 ± 0.15 ^a^
Al-Ze _(20:1)_ gelatin film	28.09 ± 0.76 ^d^	29.85 ± 0.32 ^b^	27.69 ± 0.68 ^b^
Al-Ze _(10:1)_ gelatin film	26.80 ± 0.77 ^cd^	27.68 ± 0.75 ^c^	25.14 ± 1.03 ^c^
Al-Ze _(8:1)_ gelatin film	25.20 ± 0.50 ^ab^	24.46 ± 0.18 ^d^	23.75 ± 0.75 ^d^
Al-Ze _(5:1)_ gelatin film	26.41 ± 1.21 ^b^	24.01 ± 1.02 ^d^	21.54 ± 1.68 ^e^
Al-Ze _(4:1)_ gelatin film	24.17 ± 0.89 ^a^	23.89 ± 0.52 ^d^	21.43 ± 0.45 ^e^

Note: Moisture content (MC), swelling property (SD), and water solubility (WS). ^a–e^: different lowercase letters denote significant differences (*p* < 0.05).

**Table 4:** Color change of nanoparticle gelatin films with different allicin content.

Film Sample	L*	a*	b*
Al-Ze _(20:0)_ gelatin film	88.56 ± 0.47 ^a^	−0.63 ± 0.58 ^ab^	6.02 ± 0.58 ^c^
Al-Ze _(20:1)_ gelatin film	88.26 ± 0.30 ^a^	−0.60 ± 0.05 ^b^	5.99 ± 0.42 ^c^
Al-Ze _(10:1)_ gelatin film	86.62 ± 0.23 ^b^	−0.35 ± 0.02 ^ab^	6.92 ± 0.37 ^ab^
Al-Ze _(8:1)_ gelatin film	87.47 ± 0.10 ^c^	−0.45 ± 0.02 ^b^	5.94 ± 0.53 ^c^
Al-Ze _(5:1)_ gelatin film	85.51 ± 0.34 ^d^	−0.37 ± 0.04 ^ab^	7.80 ± 0.42 ^ab^
Al-Ze _(4:1)_ gelatin film	82.77 ± 0.12 ^e^	0.18 ± 0.07 ^a^	8.74 ± 0.51 ^a^

Note: L* is the brightness; a* is red, ranging from green (−) to red (+); b* is yellow, ranging from blue (−) to yellow (+). ^a–e^: different lowercase letters denote significant differences (*p* < 0.05).

## Data Availability

All data generated or analyzed during this study are included in this published article.
